# Case Report of a Satin Guinea Pig with Fibrous Osteodystrophy That Resembles Human Pseudohypoparathyroidism

**DOI:** 10.1155/2017/1321656

**Published:** 2017-07-27

**Authors:** Miguel Gallego

**Affiliations:** Centro Veterinario Madrid Exóticos, Madrid, Spain

## Abstract

A case report of a 2-year-old female satin guinea pig with a history of dental overgrowth and lameness and radiological lesions of fibrous osteodystrophy is presented. The most relevant clinical findings were bone demineralization, high level of parathyroid hormone (PTH), normophosphatemia, normal ionized calcium, and low total thyroxine (tT4) with a normal renal function. Long-term treatment was based on teeth coronal reduction and maintaining a balanced diet. PTH measurement was performed with a kit suitable for rats to test 4 different paired samples of guinea pigs and resulted in similar results for each pair of measurements. Two kits routinely employed in dogs and cats failed in measuring PTH in guinea pig serum samples. The ionized calcium, PTH, and tT4 values, not previously reported in similar cases, were obtained. The determination of tT4 could be useful in the diagnosis of fibrous osteodystrophy in guinea pigs. The observed findings show similarity with human pseudohypoparathyroidism type Ia, a disease caused by an inactivating heterozygous mutation of the stimulatory G protein *α* subunit from the maternal genome that induces multiple hormone resistance and that courses with a syndrome called Albright hereditary osteodystrophy. Naturally occurring pseudohypoparathyroidism in animals has been reported previously only in a ferret.

## 1. Introduction

Fibrous osteodystrophy in guinea pigs is a poorly understood disease. The term Satin Guinea Pig Syndrome (SGPS) [1] has been suggested previously because it has been reported only in satin guinea pigs, and the radiological lesions are very characteristic. According to Jordan et al. (2009), 38% of satins appear to suffer from SGPS.

The SGPS usually occurs in young animals, which may present dental abnormalities, bone deformities, osteopenic lesions, pathologic fractures, elevated alkaline phosphatase, mild to moderate hypocalcemia, normo/hyperphosphatemia, low weight, and motor dysfunctions [1–5].

In one study, 8 of 11 necropsied satin guinea pigs showed interstitial nephritis, pyelonephritis, and nephrocalcinosis [2], Massop (2009) found bilaterally cystic kidneys in one satin guinea pig suffering from SGPS, and Stoffels-Adamowicz (2014) encountered glomerular basal membrane thickness and larger parathyroid glands in 7 necropsied satin guinea pigs, compared with 4 control animals. However, authors disagree on justifying the clinicopathological findings with those lesions. According to Stoffels-Adamowicz (2014), a chronic kidney disease of undetermined origin and renal secondary hyperparathyroidism (R2HP) could explain the poor bone mineralization; otherwise Schwarz and coworkers (2001) suggested nutritional secondary hyperparathyroidism (N2HP) as the cause of SGPS.

Although the SGPS appears to affect calcium metabolism, ionized calcium and PTH have not been measured previously in the affected animals, even though these parameters are crucial for the knowledge of these metabolic diseases. Parathyroid hormone has been measured previously in rats [6–8], but to the author's knowledge there is only one work concerning PTH measurement by radioimmunoassay, in the guinea pigs without SGPS [9]; unfortunately in this work PTH measurement is anecdotal and no results were published.

## 2. Case Report

A satin 2-year-old female guinea pig weighing 560 g was admitted to a private practice with anorexia, low weight, previous history of cheek teeth overgrowth and coronal reduction of 3 cheek teeth in recent months. Ten months before this visit the guinea pig was attended to for left forelimb lameness and lumbar deformity; a radiologic study was declined by the owner, and the lameness responded to meloxicam (Metacam; Boehringer Ingelheim, Sant Cugat, Spain) (0.3 mg/kg body weight, per os* q* 12 hrs, 15 days). Diet was appropriate, including nutrients rich in vitamin C.

**Figure 1:**
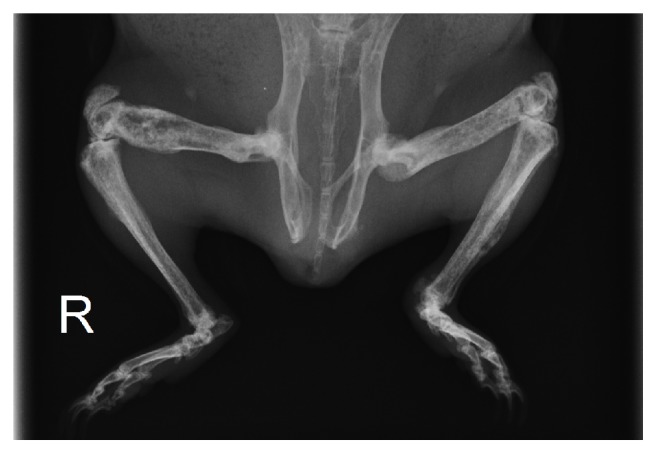
Ventrodorsal view of the rear third of a 20-month-old guinea pig with fibrous osteodystrophy. Deformation, a double cortical line, a marked trabecular pattern, and the absence of a normal medullary cavity of the long bones are observed.

A physical examination revealed total loss of mobility of the left carpal joint, malocclusion of the incisors, and cheek teeth overgrowth. The radiological study of the head showed mandibular deformation, a marked bone trabecular pattern, areas of sclerosis, and incisor malocclusion. Before sedation achieved with midazolam (Midazolam Normon; Laboratorios Normon, Tres Cantos, Spain) (0.5 mg/kg body weight, intramuscular) and butorphanol (Torbugesic; Zoetis, Alcobendas, Spain) (0.5 mg/kg body weight, intramuscular) urine was obtained by cystocentesis and blood was collected from the cranial vena cava. A hematological (Chemray 120, Rayto, Shenzhen, China) and biochemical (MS4 Vet, Melet Schloesing, Osny, France) panel, hormonal determinations, ionized calcium, and urinalysis were performed (Table 1). A whole body radiograph showed deformity, double cortical line, marked trabecular pattern, and loss of definition of the medullary cavity in virtually all of the long bones (Figure 1). Left carpal synarthrosis and misalignment of the spine at the L5-L6 level were also observed on the radiographs.

**Figure 2:**
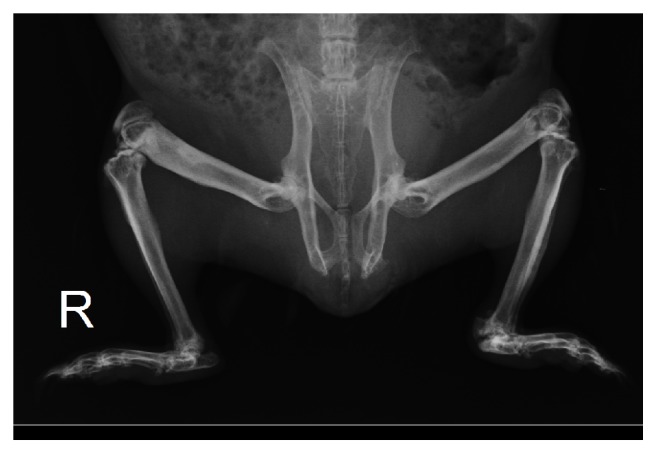
Ventrodorsal view of the rear third of a 4.5-year-old guinea pig with fibrous osteodystrophy in which hyperostosis, sclerosis, and partial remodeling are observed in previously decalcified bones. The long-term treatment was based on performing dental trimming on demand and maintaining a diet as balanced as possible.

A remarkable improvement was observed in the guinea pig's health after incisor and cheek teeth coronal reduction. As long-term management coronal reduction was performed when considered necessary (2-3 times per year), the diet was closely monitored and annual analytical controls were performed (Table 1). Three years later, the guinea pig had a good quality of life according to the owner, analytical values were stable (Table 1), and radiographs showed hyperostosis, sclerosis, and partial remodeling of the previously affected bone (Figure 2).

## 3. Material and Methods

As part of the investigation of this case, the concentration of PTH in 8 guinea pigs was measured with the consent of the owners. The 8 animals included were the guinea pig of this case report, a guinea pig that presented bilateral hydronephrosis on necropsy, and six healthy guinea pigs, according to their physical examination, hematology, and blood biochemistry values both performed as in the guinea pig of this case report. The 8 serum samples were immediately centrifuged and frozen at −18°C after extraction; 0.7 ml of these samples was sent to each of the two different veterinary diagnostic laboratories. Each one used its routine laboratory kit that is used to measure intact PTH in dogs and cats (Laboratorio Echevarne, Calle Provenza, 312 Bajos, 08037 Barcelona, Spain) (Laboklin GmbH & Co. KG, Steubenstraße 4, 97688 Bad Kissingen, Germany). Blood on EDTA of two of the 8 guinea pigs randomly selected was sent at room temperature and analyzed in less than 24 h by one laboratory (Laboratorio Echevarne, Calle Provenza, 312 Bajos, 08037 Barcelona, Spain), because the PTH levels in these samples are proven to be higher than in frozen serum [7, 15, 16]. This analysis was performed to partially discard the influence of temperature and EDTA on the results obtained in frozen plasma. All of the samples were analyzed within 24 hours of collection, which is suitable according to the literature [7, 16] and the recommendations of these laboratories for other species. Four of the previously frozen serum samples (one was the guinea pig of this case report) were sent to an experimental laboratory with experience in measuring PTH in laboratory rodents and rabbits (Department of Medicina y Cirugía Animal, Universidad de Córdoba, Campus de Rabanales, Ctra Madrid-Cádiz km 396, 14014 Córdoba, Spain). The samples remained frozen for less than 1 week. An ELISA technique validated for measurement of intact PTH in rats was employed two times in each sample.

## 4. Results

The results of PTH measurement trial and their relationship with other parameters of importance are discussed in Table 2. In the two different veterinary diagnostic laboratories (Laboratorio Echevarne, Calle Provenza, 312 Bajos, 08037 Barcelona, Spain) (Laboklin GmbH & Co.KG, Steubenstraße 4, 97688 Bad Kissingen, Germany) the concentration of PTH obtained was below the detection limit of the techniques (3-4 pg/mL). Measurements obtained in an experimental laboratory with experience in measuring PTH in laboratory rodents and rabbits (Department of Medicina y Cirugía Animal, Universidad de Córdoba, Campus de Rabanales, Ctra Madrid-Cádiz km 396, 14014 Córdoba, Spain) are shown in Table 2; a marked elevation in the concentration of serum PTH in the guinea pig of this case report compared with all other measurements of the trial was observed.

## 5. Discussion

The radiographic abnormalities observed in this case are similar to those described in the literature in guinea pigs with SGPS [1–5]. It is possible that, in the present case, the SGPS was related to injuries in left forelimb and the L5-L6 at 10 months of age.

The dental overgrowth was probably caused by dental malposition, and this was, in turn, caused by the demineralization of the alveolar bone, as observed in domestic rabbits with elevated PTH levels compared to free living rabbits [17].

Renal secondary hyperparathyroidism can occur in response to chronic renal failure, as Stoffels-Adamowicz (2014) suggested in guinea pigs with SGPS. Although a R2HP with such severe bone injuries is possible in a growing animal it would be observed in advanced chronic kidney disease associated with other indicators of impaired renal function and with a very poor health state, as occurs in other species [18–20]. Normophosphatemia, lack of azotemia, absence of anemia, and no proteinuria in the urine strip suggest normal kidney function in this case (Table 1). It is also noteworthy that, after three years, the blood and urine values did not indicate impaired renal function (Table 1).

Nutritional secondary hyperparathyroidism is caused by an inadequate Ca : P ratio in the diet, vitamin D deficiency, or vitamin D-dependent rickets [21]. The diet was similar to the remaining guinea pigs who attended the clinic daily and was based on hay, high quality pelleted food, and vegetables rich in vitamin C. Normal ionized calcium, normophosphatemia, and clinical evolution without supplements of calcium, vitamin D, or vitamin C make N2HP or scurvy unlikely in this case.

Primary hyperparathyroidism (1HP) has not been described in guinea pigs. In dogs and cats, similar to humans and rodents, 1HP always courses with hypercalcemia [21–23]. In the author's opinion normocalcemia in this guinea pig rules out 1HP.

In humans, the stimulatory G protein *α* subunit gene (GNAS) is an imprinted gene whose main product is the stimulatory G protein *α* subunit (Gs), which links numerous hormonal and transmembrane receptors to adenylyl cyclase for intracellular cAMP synthesis [24]. The rare mutations in this gene, in general, result in osteological and hormonal dysregulations mainly related to PTH, thyroid-stimulating hormone (TSH), growth hormone, ACTH, and gonadotropin releasing hormone [24]. The term pseudohypoparathyroidism (PHP) indicates a group of heterogeneous disorders whose common feature is represented by impaired signaling of various hormones (primarily PTH) that activate cAMP-dependent pathways via Gs. In humans the actual classification of the disorder is limited and includes two types: PHP type I and PHP type II. Only few cases of PHP-II have been reported and it has been hypothesized that in most cases PHP-II may be an acquired defect secondary to vitamin D deficiency [24]. The two main subtypes of PHP-I are PHP-Ia and Ib, both caused by molecular alterations of the GNAS [24–26].

PHP-Ib is characterized by renal resistance to PTH in the absence of other endocrine or physical abnormalities; these patients do not show Albright hereditary osteodystrophy. A PHP type Ic is reported, being clinically identical to PHP-Ia but without the deficiency of Gs activity in the membranes of various cell types showed in the type Ia. This difference could be explained by limitations in the assay rather than by true differences, according to some authors [24].

PHP-Ia is caused by an inactivating heterozygous mutation of the Gs from the maternal genome that courses with a syndrome called Albright hereditary osteodystrophy and resistance to various hormones [24–26]. Mutation-induced hormone resistance in PHP-Ia mainly affects PTH, TSH, and gonadotropins, with a clinical picture that depends on the severity that the mutation affects a target tissue and on which target tissues are affected [24, 25]. Resistance to PTH may not be developed by bone tissue, resulting in demineralizing/osteodystrophy effect and premature fusion of the growth plates [24–26]. Elevated levels of PTH with hypo/normocalcemia and normo/hyperphosphatemia with normal renal function are hallmarks of PHP-Ia; resistance to TSH and gonadotropins induces hypothyroidism and hypogonadism, respectively [24–26]. PHP-Ia is not a fatal disease and is usually controlled with therapy and ensuring an adequate intake of calcium and vitamin D and tends to stabilize with age [25]. In an experiment with mice, a species phylogenetically more close to the guinea pig than the man because it belongs to the same order of Rodentia, only the maternal GNAS null demonstrated resistance to PTH, suggesting that this gene, in mice, is also imprinted and that the maternal mutant phenotype is analogous to the PHP-Ia [27]. Naturally occurring pseudohypoparathyroidism in animals has been reported only in a ferret that showed lethargy, hypocalcemia, hyperphosphatemia, raised PTH, and no skeletal abnormalities; the ferret responded to calcium and vitamin D therapy [28].

The low tT4 levels observed in this satin guinea pig could reflect hypothyroidism by TSH resistance as part of PHP-Ia. In other species, low levels of tT4 are observed in euthyroid animals with no thyroid disease [29, 30], although that fact has not been described in guinea pigs [6]. Hypothyroidism rarely occurs in guinea pigs, but the clinical presentation should have been different compared to the present case report and the tT4 levels would be even lower than those observed in this guinea pig [31].

## 6. Conclusion

Bone disorders, elevated PTH levels, normocalcemia, normophosphatemia, normal renal function, and low tT4 in a young satin guinea pig with a good diet resemble the findings observed in human PHP-Ia. The determination of tT4 could be useful in the diagnosis of SGPS in guinea pigs.

In this case, the treatment was based on performing dental trimming on demand and maintaining a diet as balanced as possible. Guinea pigs with SGPS may be more sensitive than other guinea pigs to diets with an inadequate Ca : P ratio or vitamin D content.

The authors suggest that a genetic analysis of GNAS in similar cases could help to better understand this disease in guinea pigs and may be useful in human research on GNAS.

## Figures and Tables

**Table 1:** Relevant laboratory findings in a satin guinea pig with fibrous osteodystrophy.

	Day of presentation	1 year	2 years	3 years	Interval of reference
Biochemistry					
Blood urea nitrogen (mg/dL)	15	12	21	23	9–31.5^(b)^
Phosphorus (mg/dL)	4.77	3.02	2.71^*∗*^	3.06	3–7.6^(b)^
Alkaline phosphatase (U/L)	525^*∗*^	559^*∗*^	159^*∗*^	194^*∗*^	55–108^(b)^
Calcium (mg/dL)	8.4^*∗*^	7.6^*∗*^	7.3^*∗*^	9.5^*∗*^	9.6–12.4^(a)^
Creatinine (mg/dL)	0.47	0.27	0.34	0.44	0–0.87^(a)^
Urinalysis					
Urine test strip^(f)^	Normal	—	Normal	Normal	(c)
Sediment	Calcium Carbonate	—	Calcium carbonate	Calcium carbonate	Calcium carbonate, ammonium phosphate, or small amorphous crystals^(c)^
Specific gravity (refractometer)	1012	—	1011	1020	<1050^(a)^
Other					
Ionized calcium (mmol/L)	1.36	1.3	1.33	1.48	1.3–1.6^(d)^
tT4 (ug/dL)	1.3^*∗*^		1.6^*∗*^		2.26–5.82^(e)^
PTH (pg/mL)	592				—

Values not shown are within the interval of reference and include white blood cell count, red blood cell count, hematocrit, hemoglobin, mean corpuscular volume, biliary acids, alanine aminotransferase, aspartate aminotransferase, gamma glutamyl transferase, triglycerides, amylase, glucose, total bilirubin, phosphorus, albumin, and cholesterol (a, b); ^*∗*^value outside of the interval of reference; ^(a)^Wesche 2009 [10]; ^(b)^Quesenberry et al. 2004 [11]; ^(c)^Binder 2011 [12]; ^(d)^ionized calcium value obtained in-house by a sensitive electrode with a gasometer (ABL80Flex Basic, Radiometer, Bronshoj, Denmark). Reference value by Jensen et al. 2013 [13]; ^(e)^tT4 value obtained by enzyme immunoassay. Reference value by Fredholm et al. 2012 [14]; ^(f)^urine test strip (Combur 10 Test UX, Roche Diagnostics, Mannheim, Germany) measured by an automated analyser (Urisys 1100, Roche Diagnostics, Mannheim, Germany).

**Table 2:** Parathyroid hormone values measured two times in the same serum sample of 4 guinea pigs in relation to ionized calcium, creatinine, and disease.

Case	PTH (pg/dL)	PTH bis (pg/dL)	PTH above the interval of reference (25, 6–29 pg/dL)^(*∗∗*)^	iCa^(*∗*)^ (mmol/L)	Creatinine^(*∗*)^ (mg/dL)	Disease
1	592	562	Yes	1.36	0.47	SGPS of this case report
2	81	87	Yes	1.36	3.28	Bilateral hydronephrosis caused by ureteral stones (necropsy)
3	28	27.1	No	1.6	1.04	No
4	25.6	29	No	1.59	0.27	No

These eight PTH values correlate with the level of ionized calcium. In case 2, the increase in PTH is presumably due to an impaired kidney function, which contrasts with the marked increase and normal kidney function of PTH in case 1; ^(*∗*)^reference values for ionized calcium and creatinine are shown in Table 1; ^(*∗∗*)^PTH interval of reference was obtained from paired measurements of two healthy animals, cases 3 and 4.
